# Supplementary material for: Dynamic transcriptomic profiles of zebrafish gills in response to zinc supplementation
Source: BMC Genomics. 2010 Oct 11;11:553. doi: 10.1186/1471-2164-11-553 (PMC3091702; doi:10.1186/1471-2164-11-553)
Supplement: Additional file 2 — Interactive Direct Interaction Network representing the molecular interactions between zinc, copper, iron, calcium and proteins encoded by transcripts changed by zinc supplementation. Mini web-site containing index.html and hyperlinked pages in subdirectory describing a Direct Interaction Network automatically generated based on curated interactions contained within the proprietary PathwayArchitect database. Ovals represent proteins and the circles symbolize metal ions. Objects are coloured by their abundance in zebrafish at the time-point they were significantly different from the control is a scale from -4 fold (dark green) to +4 fold (dark red). Where significant differences were found at more than one time-point, the colour overlay shows expression at the first instance. Dark blue squares denote 'binding', and light blue squares 'expression'; green squares stand for 'regulation', green diamonds for 'metabolism', and green circles for 'promoter binding'. Arrow heads indicate directionality of the interaction where annotated. All nodes and edges can be further interrogated by selecting the relative area of the image. [file 1471-2164-11-553-S2.zip › PathwayArchitect Zn xs DIN/415942.html]

# TRANSPORT:

|  |  |
| --- | --- |
| Type | TRANSPORT |
| Effect | None |


---

|  |  |
| --- | --- |
| Score | 0 |


---

|  |  |
| --- | --- |
| Reference Count | 34 |


---

|  |  |
| --- | --- |
| Mechanism | Unknown |


---

|  |  |
| --- | --- |
| Reference:0 || Sentence | "As possible mechanisms for Ca2+ release from this particular pool as induced by 4-AP we examined (i) formation of Ins(1,4,5)P3 and (ii) sensitization of the Ins(1,4,5)P3-receptor/Ca(2+)release system by increasing intracellular pH." |
| PMID | 8037695 |
| Year | 1994 |
| Species | Human |
| Journal | Biochem J |
| RefScore | 1 |
| Source | PArchNLP |
  |
|  |  |


---

|  |  |
| --- | --- |
 Reference:1 || Sentence | "There is strong evidence that Ins(1,4,5)P3 stimulates Ca2+ release from intracellular stores." |
| PMID | 2850334 |
| Year | 1988 |
| Species | Human |
| Journal | J Exp Biol |
| RefScore | 1 |
| Source | PArchNLP |
  ||  |  |


---

|  |  |
| --- | --- |
 Reference:2 || Sentence | "The Ca2+-releasing actions of Ins(1,4,5)P3 are terminated by its metabolism through two distinct pathways." |
| PMID | 2850334 |
| Year | 1988 |
| Species | Human |
| Journal | J Exp Biol |
| RefScore | 0 |
| Source | PArchNLP |
  ||  |  |


---

|  |  |
| --- | --- |
 Reference:3 || Sentence | "In addition, in the same system, auranofin also failed to abolish the Ins(1,4,5)P3-induced release of Ca2+." |
| PMID | 2173626 |
| Year | 1990 |
| Species | Human |
| Journal | Biochim Biophys Acta |
| RefScore | 1 |
| Source | PArchNLP |
  ||  |  |


---

|  |  |
| --- | --- |
 Reference:4 || Sentence | "Ins(1,4,5)P3 releases Ca2+ from a specific intracellular organelle, the calciosome." |
| PMID | 2178532 |
| Year | 1990 |
| Species | Human |
| Journal | Am Rev Respir Dis |
| RefScore | 1 |
| Source | PArchNLP |
  ||  |  |


---

|  |  |
| --- | --- |
 Reference:5 || Sentence | "We believe that Ins(1,4,5)P3 induces the release of Ca2+ from intracellular storages sites which stimulates the Ca2+-dependent phospholipase A2 releasing arachidonic acid from membrane phospholipids." |
| PMID | 2939827 |
| Year | 1986 |
| Species | Human |
| Journal | Biochem J |
| RefScore | 1 |
| Source | PArchNLP |
  ||  |  |


---

|  |  |
| --- | --- |
 Reference:6 || Sentence | "Since the observations of Volpe et al. (1985) which showed that Ins(1,4,5)P3 could induce Ca2+ release from isolated terminal cisternae membranes and elicit contracture of chemically skinned muscle fibres, research has focused on the role of Ins(1,4,5)P3 in the generation of SR Ca2+ transients and in the mechanism of excitation-contraction coupling (EC-coupling). 2." |
| PMID | 8013729 |
| Year | 1994 |
| Species | Human |
| Journal | Int J Biochem |
| RefScore | 2 |
| Source | PArchNLP |
  ||  |  |


---

|  |  |
| --- | --- |
 Reference:7 || Sentence | "Ins(1,4,5)P3 induces SR Ca2+ release and the enzymes responsible for its synthesis and degradation are present in muscle tissue." |
| PMID | 8013729 |
| Year | 1994 |
| Species | Human |
| Journal | Int J Biochem |
| RefScore | 0 |
| Source | PArchNLP |
  ||  |  |


---

|  |  |
| --- | --- |
 Reference:8 || Sentence | "The Ins(1,3,4,5)P4 concentration-response curve for Ca2+ release in SH-SY5Y cells exhibited an EC50 of 2.5 microM, compared with 52 nM for Ins(1,4,5)P3, with the maximally effective concentration of Ins(1,3,4,5)P4 (100 microM) mobilizing the entire Ins(1,4,5)P3-sensitive pool." |
| PMID | 8232232 |
| Year | 1993 |
| Species | Human |
| Journal | Mol Pharmacol |
| RefScore | 1 |
| Source | PArchNLP |
  ||  |  |


---

|  |  |
| --- | --- |
 Reference:9 || Sentence | "To study whether the Ins(1,4,5)P3 receptor might be involved in thapsigargin-induced Ca2+ release, we tested the effect of heparin, a competitive Ins(1,4,5)P3 antagonist." |
| PMID | 8068001 |
| Year | 1994 |
| Species | Human |
| Journal | Biochem J |
| RefScore | 1 |
| Source | PArchNLP |
  ||  |  |


---

|  |  |
| --- | --- |
 Reference:10 || Sentence | "Taken together, our results suggest that the rapid Ca(2+)-ATPase-inhibitor-induced Ca2+ release is due to a partial activation of the Ins(1,4,5)P3 receptor in resting cells." |
| PMID | 8068001 |
| Year | 1994 |
| Species | Human |
| Journal | Biochem J |
| RefScore | 1 |
| Source | PArchNLP |
  ||  |  |


---

|  |  |
| --- | --- |
 Reference:11 || Sentence | "When the homogenate was separated into microsomes, primary granules, and secondary granules, the nonmitochondrial Ca2+ pumping and the Ins 1,4,5-P3-induced Ca2+ release occurred only in the microsomal fraction." |
| PMID | 2439542 |
| Year | 1987 |
| Species | Human |
| Journal | J Clin Invest |
| RefScore | 1 |
| Source | PArchNLP |
  ||  |  |


---

|  |  |
| --- | --- |
 Reference:12 || Sentence | "Ins(1,4,5)P3, a physiological intracellular mediator of Ca2+ release from membrane stores, did not change in concentration during these differentiation processes." |
| PMID | 1684044 |
| Year | 1991 |
| Species | Human |
| Journal | Proc R Soc Lond B Biol Sci |
| RefScore | 0 |
| Source | PArchNLP |
  ||  |  |


---

|  |  |
| --- | --- |
 Reference:13 || Sentence | "One product, Ins(1,4,5)P3, arising from the breakdown of phosphatidylinositol 4,5-bisphosphate was shown to promote the release of Ca2+ from non-mitochondrial stores in a variety of cells." |
| PMID | 3931630 |
| Year | 1985 |
| Species | Human |
| Journal | Biochem J |
| RefScore | 1 |
| Source | PArchNLP |
  ||  |  |


---

|  |  |
| --- | --- |
 Reference:14 || Sentence | "In osteoblasts and other cell types, the P2Y2 receptor is coupled to Ins(1,4,5)P3-mediated Ca2+ release from intracellular stores." |
| PMID | 9514211 |
| Year | 1998 |
| Species | Human |
| Journal | Bone |
| RefScore | 1 |
| Source | PArchNLP |
  ||  |  |


---

|  |  |
| --- | --- |
 Reference:15 || Sentence | "Ins (1,4,5)P3 at concentrations between 0.4-5.0 microM releases Ca2+ from RBC plasma membrane with an EC50 of 0.55 microM." |
| PMID | 1843204 |
| Year | 1991 |
| Species | Human |
| Journal | Receptor |
| RefScore | 1 |
| Source | PArchNLP |
  ||  |  |


---

|  |  |
| --- | --- |
 Reference:16 || Sentence | "The release was stereospecific for D-Ins(1,4,5)P3; 1 microM L-Ins(1,4,5)P3 released no more Ca2+ than the control." |
| PMID | 1843204 |
| Year | 1991 |
| Species | Human |
| Journal | Receptor |
| RefScore | 2 |
| Source | PArchNLP |
  ||  |  |


---

|  |  |
| --- | --- |
 Reference:17 || Sentence | "The potential Ca2(+)-releasing activity of the inositol tetrakisphosphates Ins(1,3,4,6)P4 and DL-Ins(1,4,5,6)P4 and the inositol pentakisphosphate Ins(1,3,4,5,6)P5 and their effect on Ins(1,4,5)P3- and DL-Ins (1,3,4,5)P4-mediated Ca2+ release were examined in permeabilized SH-SY5Y human neuroblastoma cells." |
| PMID | 1645528 |
| Year | 1991 |
| Species | Human |
| Journal | Biochem J |
| RefScore | 2 |
| Source | PArchNLP |
  ||  |  |


---

|  |  |
| --- | --- |
 Reference:18 || Sentence | "In the presence of this concentration of Ins(1,3,4,6)P4 the EC50 value for Ins(1,4,5)P3-mediated Ca2+ release increases from 0.12 +/- 0.02 microM to 2.11 +/- 0.51 microM, providing evidence that this naturally occurring inositol tetrakisphosphate may recognize and exhibit its Ca2(+)-releasing activity via the Ins(1,4,5)P3 receptor." |
| PMID | 1645528 |
| Year | 1991 |
| Species | Human |
| Journal | Biochem J |
| RefScore | 1 |
| Source | PArchNLP |
  ||  |  |


---

|  |  |
| --- | --- |
 Reference:19 || Sentence | "DL-Ins(1,3,4,5)P4, however, at its maximally effective concentration (10 microM) does not significantly affect Ins(1,4,5)P3-mediated Ca2+ release, and therefore appears to mediate its Ca2(+)-mobilizing action through a receptor distinct from that for Ins(1,4,5)P3." |
| PMID | 1645528 |
| Year | 1991 |
| Species | Human |
| Journal | Biochem J |
| RefScore | 2 |
| Source | PArchNLP |
  ||  |  |


---

|  |  |
| --- | --- |
 Reference:20 || Sentence | "Two additional Hill equations and an integration were necessary to include a putative dependence of Ins(1,4,5)P3-induced Ca2+ release on [Ca2+]i." |
| PMID | 8599644 |
| Year | 1995 |
| Species | Human |
| Journal | Biophys J |
| RefScore | 1 |
| Source | PArchNLP |
  ||  |  |


---

|  |  |
| --- | --- |
 Reference:21 || Sentence | "With the exception of sc-Ins(1,2,4,5)PS4, all the Ins(1,4,5)P3 analogues potently displaced [3H]Ins(1,4,5)P3 from its receptor in bovine adrenal cortex and were apparently potent full agonists at the Ca2+ mobilising Ins(1,4,5)P3 receptor of SH-SY5Y cells, giving respective IC50 and EC50 values of: sc-Ins(1,2,4,5)P4 (IC50 14 nM, EC50 77 nM), DL-2F-Ins(1,4,5)P3 (IC50 25 nM, EC50 105 nM), DL-Ins(1,2,4,5)P4 (IC50 26 nM, EC50 163 nM), DL-sc-Ins(1,2,4)P3 (IC50 52 nM, EC50 171 nM), compared to Ins(1,4,5)P3 (IC50 4 nM, EC50 52 nM). sc-Ins(1,2,4,5)P4 was equipotent to Ins(1,4,5)P3 for Ca2+ release making it the most potent inositol tetrakisphosphate and indeed Ins(1,4,5)P3 analogue yet characterised." |
| PMID | 8033885 |
| Year | 1994 |
| Species | Human |
| Journal | Eur J Biochem |
| RefScore | 2 |
| Source | PArchNLP |
  ||  |  |


---

|  |  |
| --- | --- |
 Reference:22 || Sentence | "A monoclonal antibody against its C-terminal domain inhibits Ins(1,4,5) P (3)-induced Ca(2+) release from ER membrane vesicles of many cell types, and an antisense-mediated knockdown of CHERP in human erythroleukemia (HEL) cells greatly impaired Ca(2+) mobilization by thrombin." |
| PMID | 12656674 |
| Year | 2003 |
| Species | Human |
| Journal | Biochem J |
| RefScore | 1 |
| Source | PArchNLP |
  ||  |  |


---

|  |  |
| --- | --- |
 Reference:23 || Sentence | "In the human T-lymphocyte cell lines Jurkat and HPB.ALL and the human monocytoid cell line U937, Ins(1,3,4,5)P4 triggers a dose-dependent release of Ca2+ from crude microsomal preparations, with a half-maximal effective concentration (EC50) of 1.2-2.3 microM." |
| PMID | 1463453 |
| Year | 1992 |
| Species | Human |
| Journal | Biochem J |
| RefScore | 1 |
| Source | PArchNLP |
  ||  |  |


---

|  |  |
| --- | --- |
 Reference:24 || Sentence | "However, in permeabilized preparations of the same cell types only Ins(1,4,5)P3 was able to release Ca2+ from intracellular stores, with EC50 values in the range 0.11-0.84 microM." |
| PMID | 1463453 |
| Year | 1992 |
| Species | Human |
| Journal | Biochem J |
| RefScore | 1 |
| Source | PArchNLP |
  ||  |  |


---

|  |  |
| --- | --- |
 Reference:25 || Sentence | "These findings show, that cultured HCASMC express G protein-coupled purinoceptors, which upon stimulation activate PLC to induce enhanced Ins(1, 4, 5)P3 production causing release of Ca2+ from internal stores." |
| PMID | 8842427 |
| Year | 1996 |
| Species | Human |
| Journal | Br J Pharmacol |
| RefScore | 1 |
| Source | PArchNLP |
  ||  |  |


---

|  |  |
| --- | --- |
 Reference:26 || Sentence | "Release of 45Ca2+ by either Tg or tBuBHQ was not affected by heparin, which totally blocked Ins(1,4,5)P3-induced Ca2+ release, and Tg was found not to affect [32P]Ins(1,4,5)P3 binding to its receptor on mixed membranes." |
| PMID | 8363562 |
| Year | 1993 |
| Species | Human |
| Journal | Biochem J |
| RefScore | 2 |
| Source | PArchNLP |
  ||  |  |


---

|  |  |
| --- | --- |
 Reference:27 || Sentence | "Ryanodine receptors (RyRs) and Ins(1,4,5)P3 receptors (Ins(1,4, 5)P3Rs) represent two multigene families of channel proteins that mediate the release of Ca2+ ions from intracellular stores." |
| PMID | 9355760 |
| Year | 1997 |
| Species | Human |
| Journal | Biochem J |
| RefScore | 0 |
| Source | PArchNLP |
  ||  |  |


---

|  |  |
| --- | --- |
 Reference:28 || Sentence | "The indications are that Ca(2+) entry through the ARC channels increases the likelihood that low concentrations of Ins(1,4,5)P(3) will trigger repetitive Ca(2+) release." |
| PMID | 14505448 |
| Year | 2003 |
| Species | Human |
| Journal | Biochem Soc Trans |
| RefScore | 1 |
| Source | PArchNLP |
  ||  |  |


---

|  |  |
| --- | --- |
 Reference:29 || Sentence | "However, Ins(1,3,4,5)P4 was found to release Ca2+ in a dose-related manner, but the response was dependent on the source of Ins(1,3,4,5)P4 used." |
| PMID | 2176465 |
| Year | 1990 |
| Species | Human |
| Journal | Biochem J |
| RefScore | 1 |
| Source | PArchNLP |
  ||  |  |


---

|  |  |
| --- | --- |
 Reference:30 || Sentence | "However, in the presence of sub-maximal concentrations of Ins(1,4,5)P3, Ca2+ release appears to be synergistic with Ins(1,3,4,5)P4, but at supramaximal concentrations not even additive effects are observed." |
| PMID | 2176465 |
| Year | 1990 |
| Species | Human |
| Journal | Biochem J |
| RefScore | 1 |
| Source | PArchNLP |
  ||  |  |


---

|  |  |
| --- | --- |
 Reference:31 || Sentence | "DAG has been shown to activate protein kinase C, whereas Ins(1,4,5)P3 induces the release of Ca2+ from an intracellular pool." |
| PMID | 3062119 |
| Year | 1988 |
| Species | Human |
| Journal | J Exp Biol |
| RefScore | 1 |
| Source | PArchNLP |
  ||  |  |


---

|  |  |
| --- | --- |
 Reference:32 || Sentence | "In permeabilized Jurkat cells, the addition of Ins(1,4,5)P3 released Ca2+; this was inhibited by heparin, whereas LPA released Ca2+ even in the presence of heparin." |
| PMID | 8912672 |
| Year | 1996 |
| Species | Human |
| Journal | Biochem J |
| RefScore | 1 |
| Source | PArchNLP |
  ||  |  |


---

|  |  |
| --- | --- |
 Reference:33 || Sentence | "Using electroporation techniques, we show that Ins(1,4,5)P(3), Ins(1,3,4, 5)P(4), and Ins(1,3,4,6)P(4) cause a rapid intracellular Ca(2+) release in resting HeLa cells and a transient increase in the frequency of ongoing Ca(2+) oscillations stimulated by histamine." |
| PMID | 10692393 |
| Year | 2000 |
| Species | Human |
| Journal | J Biol Chem |
| RefScore | 1 |
| Source | PArchNLP |
  |


---

|  |  |
| --- | --- |
